# A Fuzzy-C-Means-Clustering Approach: Quantifying Chromatin Pattern of Non-Neoplastic Cervical Squamous Cells

**DOI:** 10.1371/journal.pone.0142830

**Published:** 2015-11-11

**Authors:** Jing Rui Tang, Nor Ashidi Mat Isa, Ewe Seng Ch’ng

**Affiliations:** 1 Imaging and Intelligent System Research Team (ISRT), School of Electrical and Electronic Engineering, Universiti Sains Malaysia, Nibong Tebal, Pulau Pinang, Malaysia; 2 Advanced Medical and Dental Institute, Universiti Sains Malaysia, Bertam, Kepala Batas, Pulau Pinang, Malaysia; University of Navarra, SPAIN

## Abstract

Despite the effectiveness of Pap-smear test in reducing the mortality rate due to cervical cancer, the criteria of the reporting standard of the Pap-smear test are mostly qualitative in nature. This study addresses the issue on how to define the criteria in a more quantitative and definite term. A negative Pap-smear test result, i.e. negative for intraepithelial lesion or malignancy (NILM), is qualitatively defined to have evenly distributed, finely granular chromatin in the nuclei of cervical squamous cells. To quantify this chromatin pattern, this study employed Fuzzy C-Means clustering as the segmentation technique, enabling different degrees of chromatin segmentation to be performed on sample images of non-neoplastic squamous cells. From the simulation results, a model representing the chromatin distribution of non-neoplastic cervical squamous cell is constructed with the following quantitative characteristics: at the best representative sensitivity level 4 based on statistical analysis and human experts’ feedbacks, a nucleus of non-neoplastic squamous cell has an average of 67 chromatins with a total area of 10.827*μm* ^2^; the average distance between the nearest chromatin pair is 0.508*μm* and the average eccentricity of the chromatin is 0.47.

## Introduction

Papanicolaou-smear test is a useful screen test to detect precancerous stages of cervical cancer, thus enabling removal of intraepithelial lesions before progression into the invasive stage. Since the introduction of Pap-smear screening, mortality rate due to cervical cancer has been dramatically reduced [1,2]. In the meantime, technical advancement in slide preparation has ameliorated from conventional preparation to liquid-based preparation, overcoming the limitations of cell loss and overlapping cell morphology to a single layer of cells, thus improving specimen adequacy and further enabling better sensitivity of the test [3,4]. In Pap-smear reporting, pathologists or cytotechnologists examine the cervical epithelial cells according to the worldwide recognised reporting standard, the Bethesda System for Reporting Cervical Cytology [5]. Changes in the morphology of the cell nucleus, which are termed as malignancy-associated changes (MACs) observable under light microscope, are the prime criteria employed in this reporting standard. Changes in chromatin pattern are generally accepted as one of the MACs [6–9]. Chromatin is a complex of deoxyribonucleic acid and proteins that condenses within the nucleus [10]. Under light microscope, chromatin appears in dark and bright regions, consisting of strongly-stained heterochromatin and weakly-stained euchromatin dispersed throughout the nucleus. To report a Pap smear as negative for intraepithelial lesion or malignancy (NILM), the nuclei of squamous cells have been defined as having evenly distributed, finely granular chromatin [5,11,12].

As the definition of “evenly distributed, finely granular chromatin” for a non-neoplastic cervical squamous cell is qualitative in nature, inevitably, discrepancies between individual pathologists or cytotechnologists occur due to the subjective judgement, which would partly contribute to differences in their diagnostic accuracy [13]. To address this uncertainty in subjective judgement, it is desirable to transform those qualitatively-defined criteria to more definite quantitative criteria. Because of paramount importance of chromatin pattern as a diagnostic criterion, previous studies have been attempted to quantify the texture of nuclear chromatin. Multi-level thresholding [14–16] and region growing [17–19] techniques are generally employed. However, multi-level thresholding requires user-defined threshold values, which can highly affect the segmentation results. Furthermore, techniques based on thresholding are known to be sensitive to noise and uneven illumination. On the other hand, segmentation technique based on region growing requires predefined stopping criteria. Furthermore, the segmentation results are greatly dependent on these stopping criteria as well as the direction of the growing process. There are a few other less well known techniques, including the statistical-geometrical-features-based method [20] and the adjacency graph attribute co-occurrence matrix (AGACM) method [21]. A few studies on nuclear chromatin pattern are performed with an aid of free or commercially available software [22–24], unfortunately the details of the segmentation techniques are not known.

To overcome previous limitations in the segmentation technique of the nuclear chromatin, this study employs Fuzzy C-Means (FCM) clustering technique due to its simplicity and effectiveness in yielding promising results [25,26]. It is an unsupervised algorithm, in addition to its robustness for ambiguity and its ability which always converges. By employing a reasonably defined number of clusters, this technique would enable the segmentation of chromatin from cervical squamous cell nuclei to be achieved at different sensitivity levels, thus stimulating the differences in interpretation threshold of pathologists or cytotechnologists in their subjective judgment of “evenly distributed, finely granular chromatin”. To our best knowledge, study on segmentation of chromatin pattern of cervical squamous cells captured from ThinPrep slides, a liquid-based preparation as compared to conventional smear slides, using the clustering technique, has yet to be performed. At the end of this study, a model representing the chromatin distribution of a non-neoplastic cervical squamous cell will be presented.

## Materials and Methods

### 2.1. Cervical Squamous Cell Image Acquisition

The study was approved by the Human Research Ethics Committee of Universiti Sains Malaysia with the reference code: USMKK/PPP/JEPeM[217.4(2.6)]. Human Research Ethics Committee of Universiti Sains Malaysia is listed under the Office for Human Research Protections (OHRP), United States Department of Health & Human Services. The Federal-wide Assurance (FWA) identification number is FWA00007718 and the Institutional Review Board (IRB) number is IRB00004494. The ThinPrep slides were obtained from Penang General Hospital, Malaysia. No consent was given since the patients' information is blinded. The ThinPrep slides were received without any patients' information. The cervical cell images were captured and analysed anonymously. These slides had been previously screened by cytotechnologists and formally reported as “negative for intraepithelial lesion or malignancy” by pathologists. They were reviewed and cervical squamous cell images were captured by a pathologist, using an Olympus BX43F clinical microscope mounted with a video camera. Oil immersion with 100x objective is used. The single cell image is manually cropped into the size of 500 x 500 pixels to obtain its nucleus. A total of 150 cropped test images (S1 File) are used in this study.

### 2.2. Methodology

The processing of the cervical squamous cell images consists of three stages, i.e. the pre-processing stage, the chromatin segmentation stage and the feature extraction stage. During the pre-processing stage, the input colour image with the size of 2048 x 1536 pixels is initially cropped at a size of 500x500 pixels to obtain the nucleus. The colour nucleus image is then converted into gray scale image. The contrast of the image is enhanced by stretching its input histogram to occupy the entire dynamic intensity range.

With the cropped nucleus, Fuzzy C-Means (FCM) clustering technique is applied to segment the chromatin. FCM clustering technique is first proposed by Bezdek [27]. The objective function of FCM algorithm is defined as [28]:

$$W_{m}=\sum_{i=1}^{c}\sum_{j=1}^{n}{\left(\mu_{ij}\right)}^{m}{∥x_{j}-v_{i}∥}^{2}$$ (1)

with *μ*_*ij*_ ∈[0,1], $\sum_{i=1}^{c}\mu_{ij}=1$, $0\leq \sum_{j=1}^{n}\mu_{ij}\leq n$

Parameter *μ*_*ij*_ is the degree of membership of *x*_*j*_ belonging to the *c*-th cluster. Parameter *m*, a scalar value greater than one, is the weighting exponent which controls the amount of fuzziness of the resulting partitions. The operator ∥∥ represents the Euclidean norm. Parameter *v*_*i*_ represents the set of cluster centroids. FCM is performed by minimizing Eq (1) through equations updating of membership function *μ*_*ji*_ and cluster centroid *v*_*i*_ as presented in Eqs (2) and (3). The number of cluster is defined as the value of the intensity, which coincides with the peak of the histogram of the nucleus. Details on assigning the initial cluster number are further illustrated in S2 File with S1, S2, S3, S4 and S5 Figs. Values of parameters in FCM are defined as in Table 1. Parameter testing has been performed for *m*. Complexity of FCM in segmenting the chromatin is justified as well through the computational time. Results and analyses of parameter testing and complexity are presented in S3 File with S1 to S9 Tables and S4 File with S10 Table respectively.

**Table 1:** Parameter setting in FCM.

Parameter	Value
*m*	2.0
Maximum Number of Iteration	100
Minimum Amount of Improvement	1E-05

$$\mu_{ij}=\frac{1}{\sum_{k=1}^{c}{\left(\frac{{∥x_{j}-v_{i}∥}^{2}}{{∥x_{j}-v_{k}∥}^{2}}\right)}^{\frac{1}{m-1}}}$$ (2)

$$v_{i}=\frac{\sum_{j=1}^{n}{\left(\mu_{ij}\right)}^{m}x_{j}}{\sum_{j=1}^{n}{\left(\mu_{ij}\right)}^{m}}$$ (3)

As discussed in the previous section, there is no exact term to describe the exact intensity level for chromatin. Questions arise such as to which extend we can define a dark region in the nucleus as chromatin? How ‘dark’ a region can be considered as chromatin? Thus, an attempt is made here where several levels of intensity threshold, which is known as the sensitivity level in this paper, is proposed. In this study, the sensitivity level of the chromatin detection can be defined by user. The sensitivity level defines the intensity threshold for chromatin segmentation. Here, for the purpose of initial study, the distribution of chromatin is observed for five sensitivity levels. From the segmented image obtained from FCM clustering, the intensities of all pixels are sorted in the ascending order. The five most minimum intensity values at sensitivity level *k*, which are the five lowest intensity values obtained from the segmented image, are taken as the intensity threshold *t*_*k*_, where *t*_1_ < *t*_2_ < *t*_3_ < *t*_4_ < *t*_5_ and *t*_*k*_ ∈[0.255].

## Simulation Results

**Fig 1:**
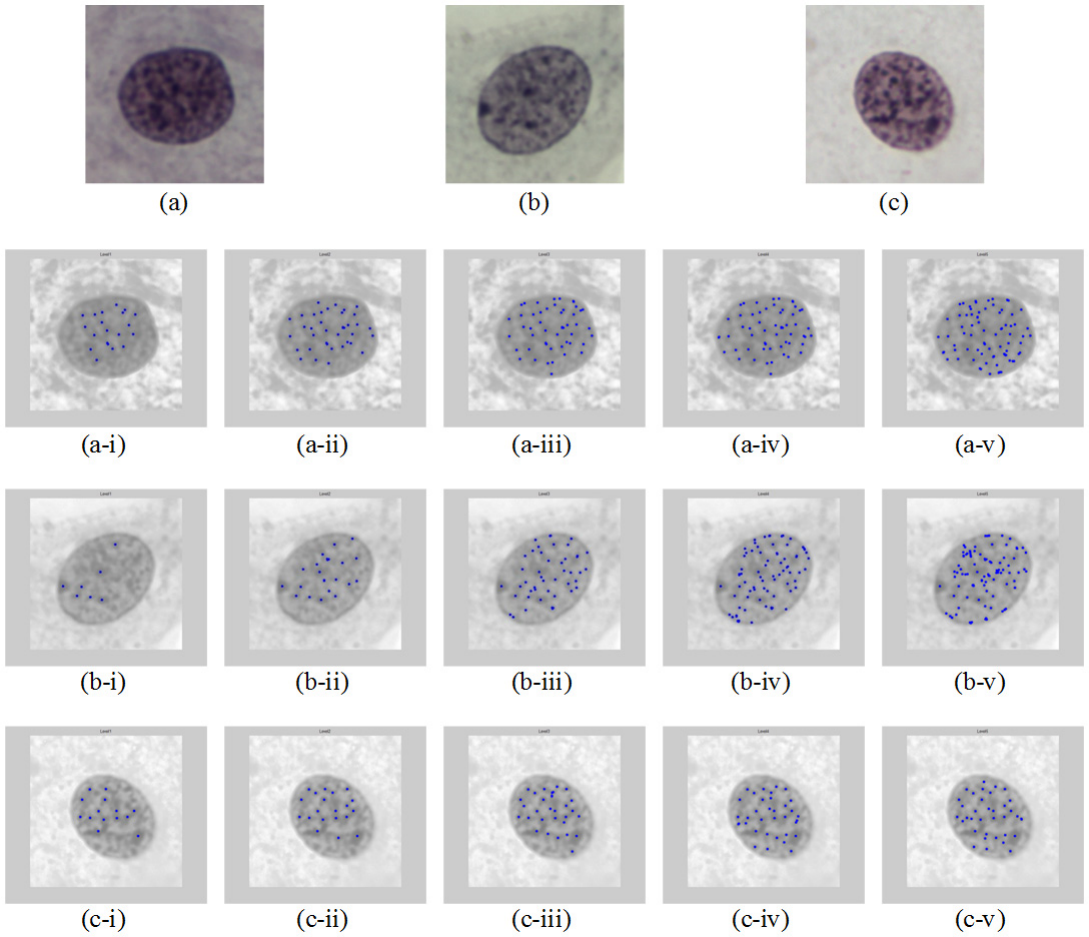
Chromatin appeared in alternating dark and bright regions as demonstrated in Test Image: (a) 1; (b) 2 and (c) 3. Test Image 1 segmented at sensitivity level =: (a-i) 1, (a-ii) 2, (a-iii) 3, (a-iv) 4, (a-v) 5; Test Image 2 segmented at sensitivity level =: (b-i) 1, (b-ii) 2, (b-iii) 3, (b-iv) 4, (b-v) 5 and Test Image 3 segmented at sensitivity level =: (c-i) 1, (c-ii) 2, (c-iii) 3, (c-iv) 4, (c-v) 5.

Simulation results of three randomly selected test images are demonstrated in Fig 1. The number of detected chromatin is increased with the increment of the sensitivity level. The dotted points represent the centres of the chromatin regions detected. Three measurements are computed for better understanding on the spread of the data, which are the distance between two nearest chromatin pair, the area of the chromatin and the eccentricity of the chromatin. For each image, the average value of each measurement is computed. For example, if a test image contains ten detected chromatin regions at sensitivity level 1, the average values of the size (area) of the chromatin, the average distance between all the two nearest chromatin pair and the average eccentricity for all the chromatin regions are computed.

Consider an input cervical image, *j* segmented at sensitivity level *k*, there are *n* chromatin regions detected. The total area of the chromatin regions can be defined as

$$A_{jk}=a_{1jk}+a_{2jk}+\cdots +a_{njk}$$ (4)

$$=\sum_{i=1}^{i=n}a_{ijk}$$ (5)

where *a*_*ijk*_ is the area of chromatin region *i* in the image *j* at sensitivity level *k*.

The average chromatin region can be represented as

$${\bar{a}}_{jk}=\frac{A_{jk}}{n}$$ (6)

The overall average area of a chromatin region in 150 test images can be defined as

$${\bar{\bar{a}}}_{jk}=\frac{\sum_{j=1}^{150}A_{jk}}{\sum_{j=1}^{150}n_{jk}}$$ (7)

For every chromatin region, the centre of the region is computed to find the distance between every chromatin pair, *d*_*njk*_.

$$d_{njk}=\left[\begin{array}{cccc} d_{\left(1)-(1\right)} & d_{\left(1)-(2\right)} & \ldots & d_{\left(1)-(n\right)}\\ d_{\left(2)-(1\right)} & d_{\left(2)-(2\right)} & \ldots & d_{\left(2)-(c\right)}\\ \vdots & \vdots & \cdots & \vdots \\ d_{\left(n)-(1\right)} & d_{\left(n)-(2\right)} & \ldots & d_{\left(n)-(n\right)} \end{array}\right]$$ (8)

where *d*_(1)-(1)_ is the distance between chromatin regions 1 and 1 and *d*_(1)-(*n*)_ is the distance between chromatin regions 1 and *n*.

The nearest chromatin pair is obtained with

$$d_{jk}=\text{min}\left(d_{njk}\right)$$ (9)

The average distance of the nearest chromatin pair for an image *j* at sensitivity level *k* is defined as

$${\bar{d}}_{jk}=\frac{\sum_{i=1}^{n}\text{min}\left(d_{ijk}\right)}{n}$$ (10)

The overall average distance of the nearest chromatin pair of 150 test images at sensitivity level *k* can be represented by

$${\bar{\bar{d}}}_{jk}=\frac{\sum_{j=1}^{150}{\bar{d}}_{jk}}{150}$$ (11)

In addition to the chromatin size and distance between chromatin pair, the eccentricity of each chromatin, E is computed. Eccentricity is the ratio of the length of major axis and the length of minor axis. It is invariant for geometric transformations and can be defined as

$$\text{E}=\frac{\text{Length of major axis }}{\text{Length of minor axis }}$$ (12)

The total eccentricity of the input image, *j* at sensitivity level *k* with *n* chromatin regions can be defined as

$${\text{E}}_{jk}=\varepsilon_{1jk}+\varepsilon_{2jk}+\cdots +\varepsilon_{njk}$$ (13)

$$=\sum_{i=1}^{i=n}\varepsilon_{ijk}$$ (14)

where *ε*_*ijk*_ is the eccentricity of chromatin region *i* in the image *j* at sensitivity level *k*.

The average eccentricity of the chromatin for image *j* can thus be defined as

$${\bar{\varepsilon }}_{jk}=\frac{{\text{E}}_{jk}}{n}$$ (15)

At sensitivity level *k*, the overall average eccentricity for 150 test images is defined as

$${\bar{\bar{\varepsilon }}}_{jk}=\frac{\sum_{j=1}^{150}{\bar{\varepsilon }}_{jk}}{150}$$ (16)

**Fig 2:**
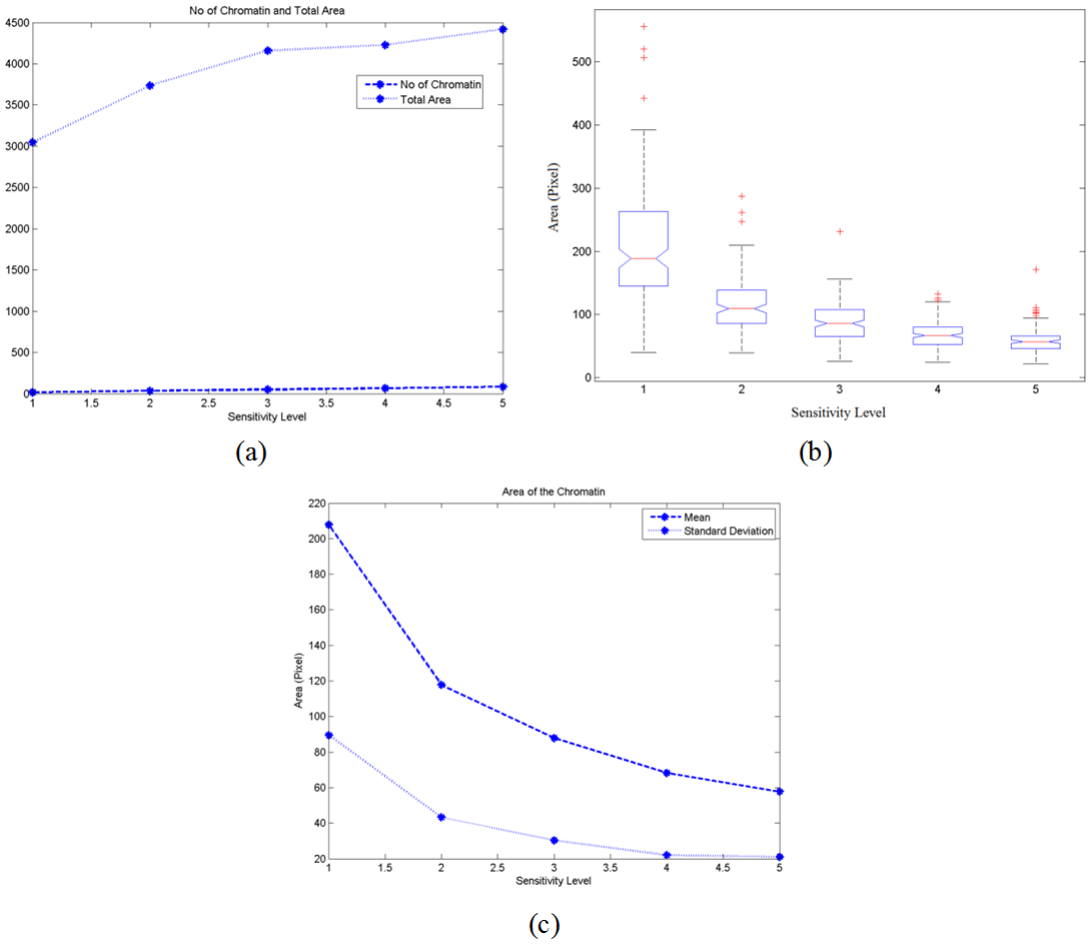
(a) Plot of the total number of chromatin detected with the total area as the sensitivity level increases; (b) boxplot of 150 test images for the average of area of the chromatin and (c) plot of the mean and standard deviation of the average area of chromatin for 150 test images.

Fig 2 presents the analyses for the chromatin area. Fig 2(a) shows the total number of chromatin detected,$\sum_{j=1}^{150}n_{jk}$ and the total area computed of chromatin, $\sum_{j=1}^{150}A_{jk}$ for all the 150 test images at sensitivity level *k*. Fig 2(b) on the other hand demonstrates the boxplot of the average area of the chromatin for the 150 test images. The mean and the standard deviation of the average area of chromatin for 150 test images are plotted in Fig 2(c).

**Fig 3:**
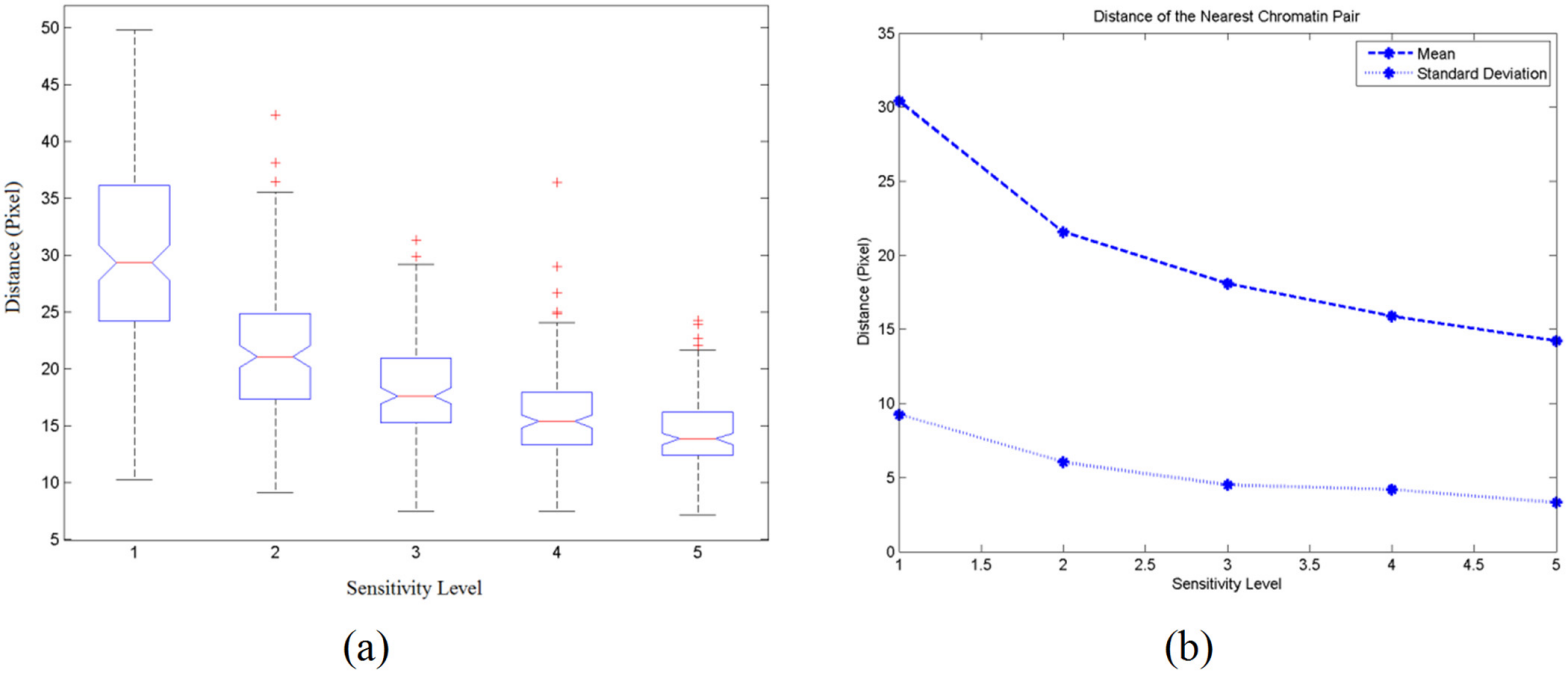
(a) Boxplot of 150 test images for the average distance of the nearest chromatin pair and (b) plot of the mean and standard deviation of the average distance of the nearest chromatin pair for 150 test images.

**Fig 4:**
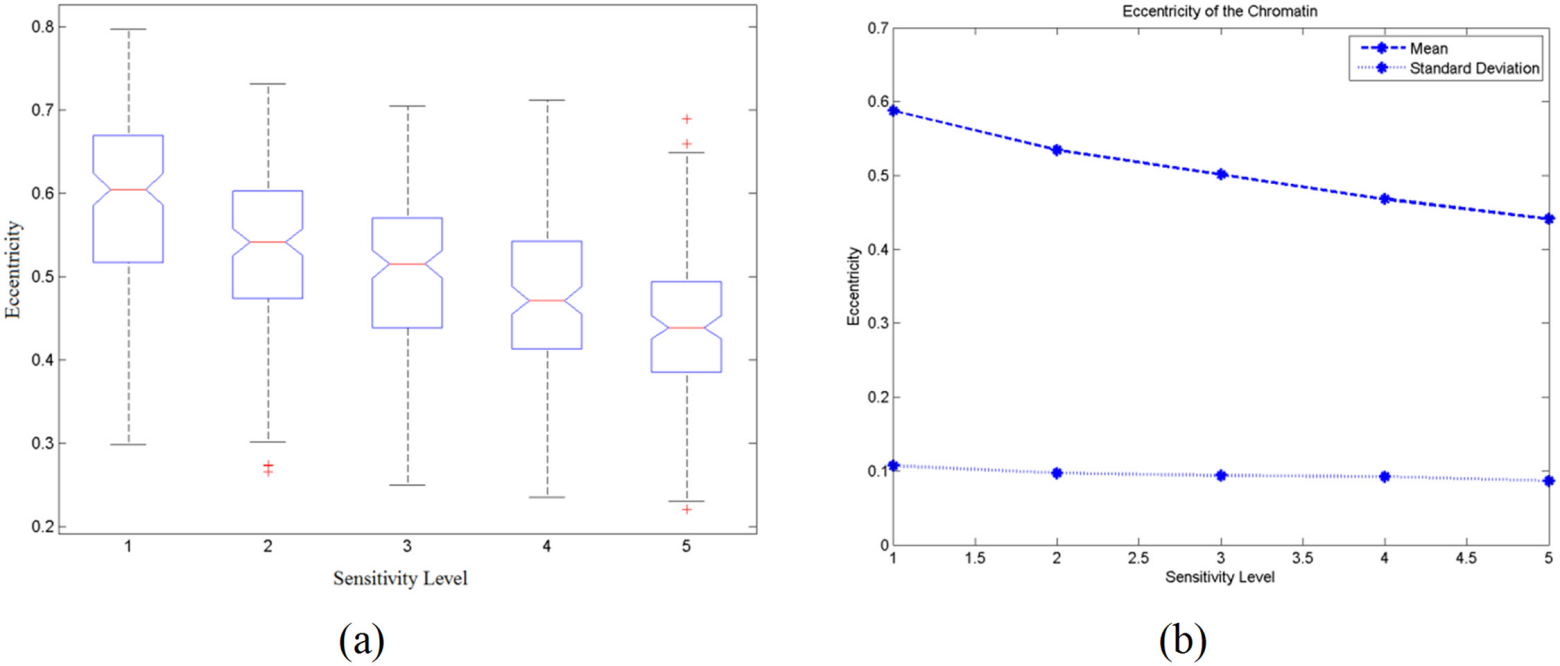
(a) Boxplot of 150 test images for the average eccentricity and (b) plot of the mean and standard deviation of the average eccentricity for 150 test images.

For the measurement of the distance of the nearest chromatin pair, the boxplot of the 150 test images is shown in Fig 3(a). The mean and standard deviation of the average distance of the nearest chromatin pair for these test images are shown in Fig 3(b). Fig 4 demonstrates the changes in shape of the chromatin detected. Fig 4(a) shows the boxplot of the average eccentricity for 150 test images and Fig 4(b) shows the mean and standard deviation of the average eccentricity values of these images.

For further analysis on the data, Friedman test is performed to test for differences between the five sensitivity level groups. For the both the average area and the average distance of the nearest chromatin pair, the tests reveal a statistically significant difference result, with *p*-value less than α. Null hypothesis stating that there is no differences between the sensitivity levels is rejected. Post hoc test is performed to further identify which sensitivity levels differ from which other sensitivity levels in the measurement of average area and average distance of the nearest chromatin pair. Summary from the results of the post hoc analysis with Holm’s and Shaffer’s procedures are presented in Table 2. Details of the post hoc test are presented in the supplementary material. Since comparison of all five sensitivity levels at four different amount of fuzziness reported significantly difference except for a pair of sensitivity level, Table 2 presents the sensitivity levels at which there are no significant difference for both the analysis of average area of chromatin and average distance of the nearest chromatin pair. Sensitivity levels other than those presented in Table 2 demonstrated adjusted *p*-values less than 0.05.

**Table 2:** Summary of N × N comparisons of five sensitivity levels that do not have significant difference.

Amount of Fuzziness, *m*	Level vs. Level	
	Average Area of Chromatin	Average Distance of the Nearest Chromatin Pair
1.2	[4] vs. [5]	[3] vs. [4]
2.0	[4] vs. [5]	[4] vs. [5]
3.0	[4] vs. [5]	[4] vs. [5]
4.0	[4] vs. [5]	[4] vs. [5]

**Fig 5:**
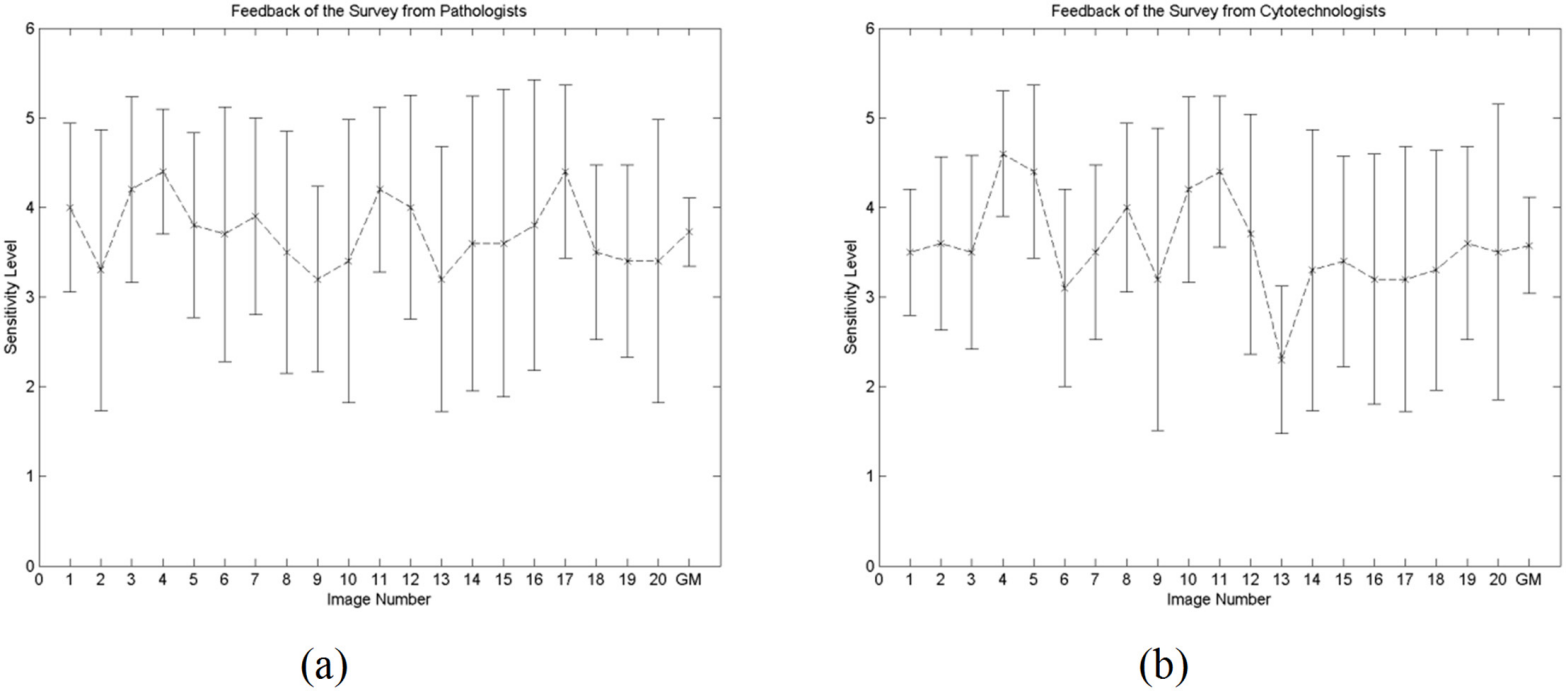
Mean and standard deviation of the chosen sensitivity levels based on image number from (a) pathologists and (b) cytotechnologists. The grand average sensitivity levels are shown as the final plot (i.e. labelled as ‘GM’).

From Table 2, for the average distance of the nearest chromatin pair, only sensitivity level 3 and 4 reported analysis that has no statistical difference. For all the other analysis, sensitivity levels 4 and 5 reported analysis that is not statistically different. As the amount of fuzziness changes from 2.0 to 4.0, there is no difference in the post hoc results. Therefore, in this study, the amount of fuzziness employed is 2.0. In addition to statistical analyses, a survey was conducted to justify the most representative sensitivity levels of human experts. The feedbacks from both the pathologists and cytotechnologists reported the ‘reality visual perception’ as compared to the findings from the statistical analyses. Ten pathologists and ten cytotechnologists participated in the survey. The survey consists of 20 questions, each presented with five sensitivity levels of chromatin detection. The human experts independently selected the images which best suit the chromatin as perceived. The mean and standard deviation of the sensitivity levels chosen for pathologists and cytotechnologists are demonstrated in Fig 5(a) and 5(b) respectively. The grand average sensitivity level for pathologists and cytotechnologists are 3.725±0.380 and 3.575±0.537 level respectively. The statistically selected sensitivity level matched the visual perception of pathologists and cytotechnologists. The grand average sensitivity levels of 3.725 and 3.575 reveal that sensitivity level 4 is the most representative level for chromatin detection.

## Discussions

Changes in the morphology of the cell nucleus are recognized as one of the crucial phenomena associated with neoplastic transformation [29]. These malignancy-associated changes (MACs) in the cell nucleus, particularly changes in the chromatin pattern, are employed as diagnostic criteria in the Bethesda System for Reporting Cervical Cytology for precancerous and cancerous diagnostic categories [5]. Separating the non-neoplastic category, i.e. negative for intraepithelial lesion or malignancy (NILM), from the neoplastic categories, chromatin pattern of cervical squamous cells of NILM has been defined in several literatures as in Table 3.

**Table 3:** Description on criteria of chromatin to report a case as NILM.

Reference	Description
[7]	The pattern is finely granular.
[8]	Usually, chromatin pattern of nucleus of normal cell is fine.
[11]	The chromatin is finely and uniformly granular.
[15]	Mild hyperchromasia may be present, but the chromatin structure and distribution remain uniformly finely granular.

It is apparent from Table 3 that the criteria of the reporting standard are qualitative in nature. Pathologists or cytotechnologists acquire their diagnostic skills through observation of numerous slides based on these qualitatively defined criteria. It is inevitable that discrepancies between individual pathologists or cytotechnologists occur due to the subjective judgement based on these criteria, which might lead to different diagnostic results. Therefore, there is a need to transform those qualitatively-defined criteria to more definite quantitative criteria. A computer-aided tool which is capable to analyse and quantify the characteristics light microscope changes would be useful in the process of transition from qualitative criteria to quantitative criteria.

Various attempts have been made to investigate the chromatin pattern in the cell nucleus. Rowinski et al. [30] measured the area of the chromatin of the lymphocytes using the Image Analysing Computer Quantimet based on multi-level thresholding. Smeulders et al. [31] segmented the chromatin of the cervical cells where the size of the segmented region is restricted by the lowest gray level and the lowest gray level gradient. Similar technique which limits the growing based on a fixed percentage of the nuclear area is then proposed [32]. Young et al. [33] on the other hand measured the heterogeneity, granularity, condensation and margination of chromatin by dividing the nucleus image into three categories based on thresholding. Murata et al. [34] employed 2-dimentional and higher texture analysis to analysis chromatin pattern of thyroid tumor cells. Jingu et al. [15] measured the gradient of the staining intensity from the center to the border of the nucleus as an index of the chromatin distribution of cervical squamous epithelial cells. These previous works have emphasized the usefulness of chromatin pattern in diagnostic. Although the above mentioned techniques tried to refine the descriptive terms for chromatin pattern such as homogeneity, clumping, and granularity used by the pathologists or cytotechnologists, they do not take into consideration the issue of different judgments by individual pathologist or cytotechnologist due to different sensitivities in visual perception of chromatin.

To imitate the human diagnostic behavior, this study proposed different sensitivity levels for the segmentation of chromatin pattern to represent the potential view of individual pathologist. The aim of this study is to quantify the statement “evenly distributed, finely granular chromatin” and hence build a model for the chromatin distribution of non-neoplastic cervical squamous cell. For chromatin regions detected at each level, three parameters are computed: the area of chromatin, the distance between two nearest chromatin pair, and the eccentricity of chromatin. Firstly, to quantify the so called “finely granular chromatin”, the area of the chromatin is computed based on the total number of pixels which are connected in neighbourhood and have the same intensity values in the segmented image. The area of the chromatin would reflect the degree of fineness of chromatin quantitatively. Secondly, to quantify the so-called “evenly distributed chromatin”, the Euclidean distance between two centres of the nearest chromatin pair is computed. The average distance of the nearest chromatin pair with minimum standard deviation would reflect the degree of even distribution quantitatively. Thirdly, the eccentricity of the chromatin is computed to investigate the change in the shape of the chromatin detected as the sensitivity level increases, which is another aspect of chromatin pattern that has yet to be explored.

From Fig 2(a), the increment rate of the average number of chromatin detected is greater than the rate for the average total area of the chromatin. Thus, the average area of the chromatin decreasing with the increasing of sensitivity levels as shown in Fig 2(b) and 2(c). When the sensitivity level increases, more chromatin can be detected and this might result in the generation of more overlapping and combination of regions. The elimination of overlapping regions and replacement of these regions with the regions detected at lower sensitivity level reduces the rate of increment of the average total area. Details of the issues on regions overlapping and combination can be found in the supplementary material. The boxplot of the area for 150 test images in Fig 2(b) shows the decreasing trend in the median value of the size of the chromatin as the sensitivity level increases. The interquartile range becomes smaller as the sensitivity level increases. This indicates that when more chromatin are detected, their average size become similar. The standard deviation decreases with the increment of sensitivity level. It could be implied from these stimulation results that at a sufficient level of sensitivity, i.e. level 3 and above, the size of the chromatin detected would have less fluctuation; this would result in almost similar fine granular chromatin pattern for the visual perception of most pathologists and cytotechnologists.

From Fig 3(a) and 3(b), the distance between the nearest chromatin pair decreases as the sensitivity level increases. When more chromatin is detected at higher sensitivity level, the distance between all nearest chromatin pair becomes shorter. Although the distance between the nearest chromatin pair decreases, the standard deviation decreases at lower rate where it does not vary significantly as compared to the area. This indicates that the sensitivity level less affects the chromatin distribution in terms of their distances. As the sensitivity level increases, the distance between the nearest chromatin pair has little difference. Thus, it can be concluded that the chromatin distribution of the cervical nucleus image always appeared to be evenly distributed, provided the amount of chromatin detected is at a sufficient level. A pathologist might perceive different amount of chromatin from another pathologist. From the observation on the distance between the nearest chromatin pair, these pathologists will eventually observed the similar distribution of chromatin patterns because the chromatin appeared to be evenly distributed regardless the sensitivity level.

The eccentricity values demonstrated in Fig 4(a) and 4(b) show that the shape of the chromatin lies within the range of circle and ellipse. As the sensitivity level increases, the chromatin shape becomes more round with their eccentricity value getting closer to zero. The interquartile range for the eccentricity of every sensitivity level is similar with the median value decreases as shown in Fig 4(a). The standard deviation appeared to be constant regardless the changes in sensitivity level. This shows that as the sensitivity level increases, even though the shape of the chromatin detected has increasing roundness, the eccentricity values of all the chromatin regions in an image show little difference among each other.

**Fig 6:**
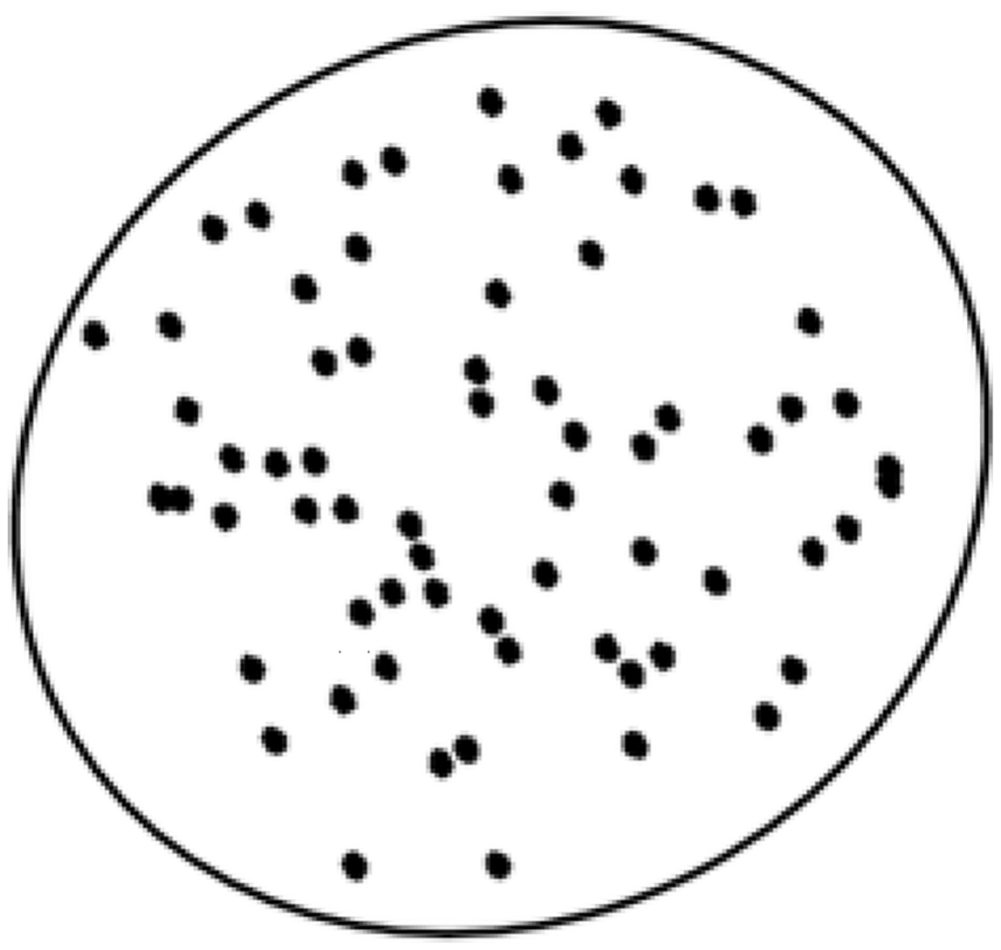
Simulated model of ‘evenly distributed, fine granular chromatin’ (i.e. at sensitivity level = 4).

From the statistical analysis, sensitivity level 4 appeared to be the most sufficient level to represent the distribution of the chromatin pattern for non-neoplastic cervical squamous cell. From Table 2, the chromatin pattern at both sensitivity levels 4 and 5 has insignificant difference for both chromatin size and the distance between the nearest chromatin pair as the amount of fuzziness changed from 2.0 to 4.0. The steady trend in the standard deviation of these two sensitivity levels for the measurement of the chromatin area and the distance between the nearest chromatin pair could also be regarded as equivalent to the criteria ‘evenly distributed, fine granular chromatin’ for the classification of non-neoplastic cervical squamous cells. Therefore, statistically, the most representative sensitivity level is 4. Cross-checking this statistically selected sensitivity level, the visual reality perception in the form of survey of human experts also returned similar grand average sensitivity levels of 3.725 and 3.575, validating the sensitivity level 4 as the most representative level for model construction of chromatin pattern. With the simulation values as shown in Table 4, we develop the model for the distribution of chromatin pattern based on the proposed technique. The model is shown in Fig 6.

**Table 4:** Parameters for simulated model of ‘evenly distributed, fine granular chromatin’ at sensitivity level = 4.

Average Number of Chromatin	Total Area of Chromatin (Pixel)	Average Area per Chromatin (Pixel)	Average Distance between the Nearest Chromatin Pair (Pixel)	Average Eccentricity per Chromatin
67	4229.43(equivalent to 10.827*μm* ^2^)	63.44(equivalent to 0.162*μm* ^2^)	15.88(equivalent to 0.508*μm*)	0.47

## Conclusion

In this study, we have quantified the criteria ‘evenly distributed, fine granular chromatin’ for the chromatin pattern of non-neoplastic cervical squamous cell. The tool which implements the Fuzzy C-Means clustering technique segment the chromatin from cervical squamous cell nuclei at different sensitivity levels and thus imitating the different chromatin detection sensitivity of individual pathologist or cytotechnologist based on his/her experience and understanding on the subjectively-defined criteria. A model representing the distribution of chromatin pattern for non-neoplastic cervical squamous cell is developed with the following quantitative features: a nucleus of non-neoplastic squamous cell has an average of 67 chromatins with a total area of 10.827*μm* ^2^, the average distance between the nearest chromatin pair is 0.508*μm* and the average eccentricity of the chromatin is 0.47. As an initial effort to quantify the criteria in a definite way, the tool could be useful to the pathologists as it can be installed in laboratories and hence eliminates the discrepancies of diagnostic due to the ambiguity of defining the criteria. For future improvement, more sample cervical squamous cells could be included for a better representation of chromatin features and we will further extend our work to cases of low grade and high grade squamous intraepithelial lesion.

## Supporting Information

S1 Fig The histogram of the image of cropped nucleus. (TIF)

S2 Fig The sensitivity level and the intensity threshold can be imagined as the contour of a mountain. (TIF)

S3 Fig Overlapping region. (a) Detected regions at lower sensitivity level; (b) detected regions at higher sensitivity level; (c) overlapping of regions and (d) preserving the regions detected at lower sensitivity level for overlapping regions and obtain final segmentation results.
(TIF)

S4 Fig Combination of two chromatin regions: (a) Chromatin regions at lower sensitivity level; (b) Chromatin region at higher sensitivity level and (c) The centres of the chromatin regions when regions A, B and C overlap. (TIF)

S5 Fig Flowchart of chromatin segmentation. (TIF)

S1 File Dataset. (RAR)

S2 File Defining Cluster Number. (DOCX)

S3 File Parameter Testing for Amount of Fuzziness. (DOCX)

S4 File Complexity Analysis of FCM. (DOCX)

S1 Table *p-values* of Friedman Test. (DOCX)

S2 Table Adjusted *p-values* for N × N comparisons of five sensitivity levels for the area of chromatin with m = 1.2. (DOCX)

S3 Table Adjusted *p-values* for N × N comparisons of five sensitivity levels for the area of chromatin with m = 2.0. (DOCX)

S4 Table Adjusted *p-values* for N × N comparisons of five sensitivity levels for the area of chromatin with m = 3.0. (DOCX)

S5 Table Adjusted *p-values* for N × N comparisons of five sensitivity levels for the area of chromatin with m = 4.0. (DOCX)

S6 Table Adjusted *p-values* for N × N comparisons of five sensitivity levels for the distance between nearest chromatin pair with m = 1.2. (DOCX)

S7 Table Adjusted *p-values* for N × N comparisons of five sensitivity levels for the distance between nearest chromatin pair with m = 2.0. (DOCX)

S8 Table Adjusted *p-values* for N × N comparisons of five sensitivity levels for the distance between nearest chromatin pair with m = 3.0. (DOCX)

S9 Table Adjusted *p-values* for N × N comparisons of five sensitivity levels for the distance between nearest chromatin pair with m = 4.0. (DOCX)

S10 Table Average computational time for 150 test images. (DOCX)
