# Supplementary figures and images for: A Fuzzy-C-Means-Clustering Approach: Quantifying Chromatin Pattern of Non-Neoplastic Cervical Squamous Cells
Source: PLoS One. 2015 Nov 11;10(11):e0142830. doi: 10.1371/journal.pone.0142830 (PMC4641582; doi:10.1371/journal.pone.0142830)

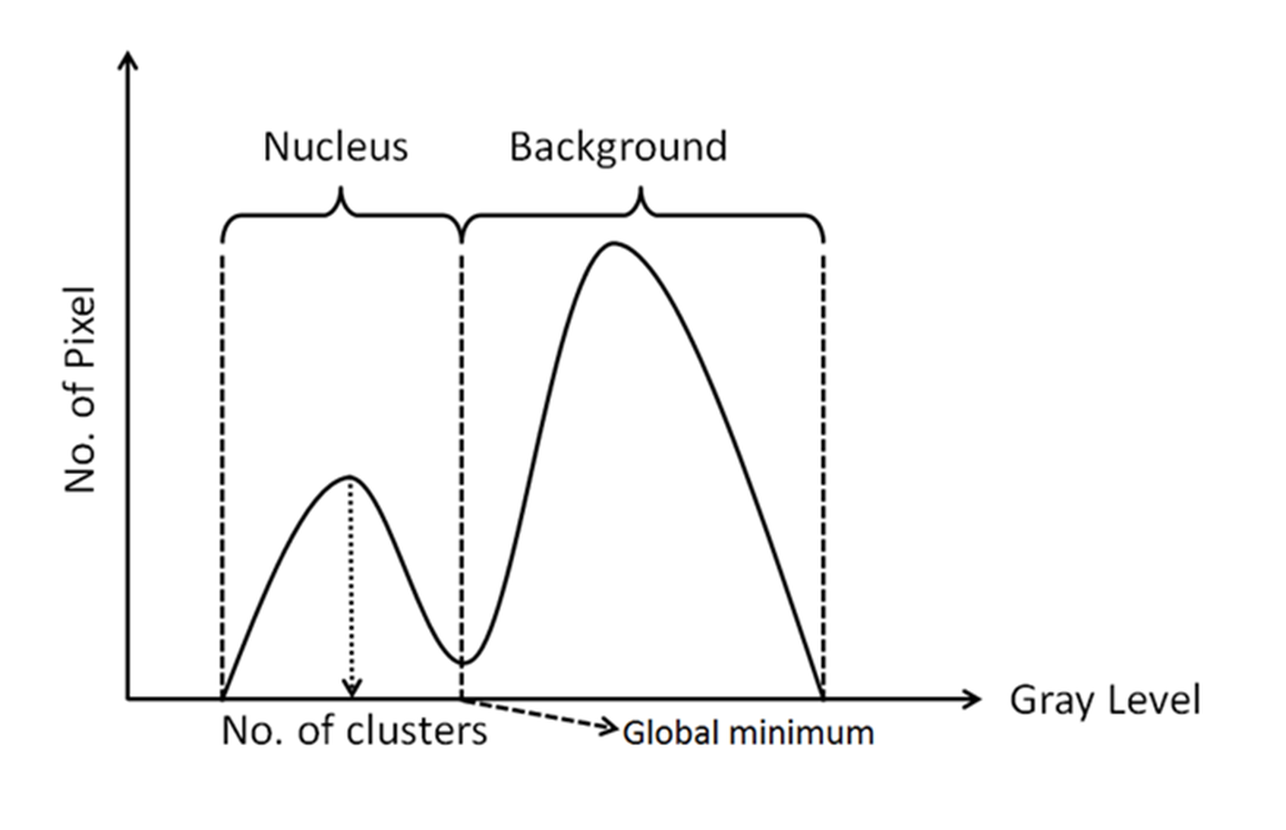

Supplement: S1 Fig — (TIF) [file pone.0142830.s001.tif]

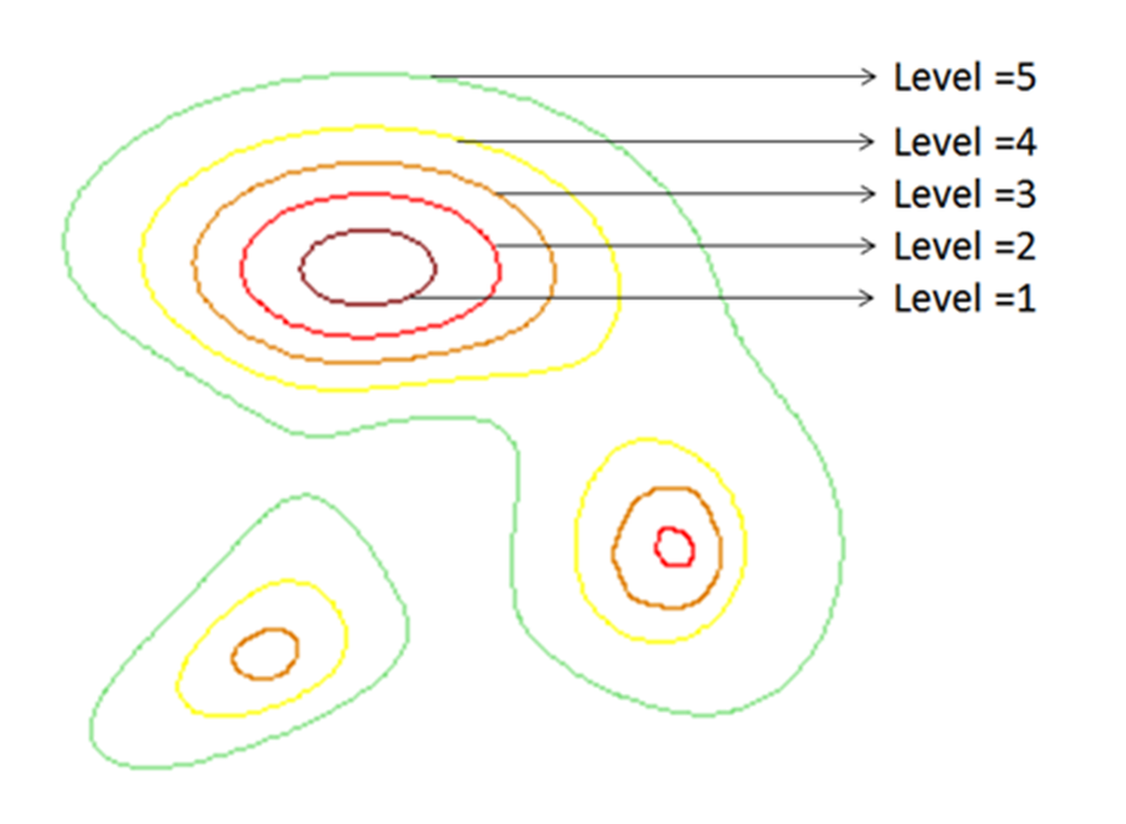

Supplement: S2 Fig — (TIF) [file pone.0142830.s002.tif]

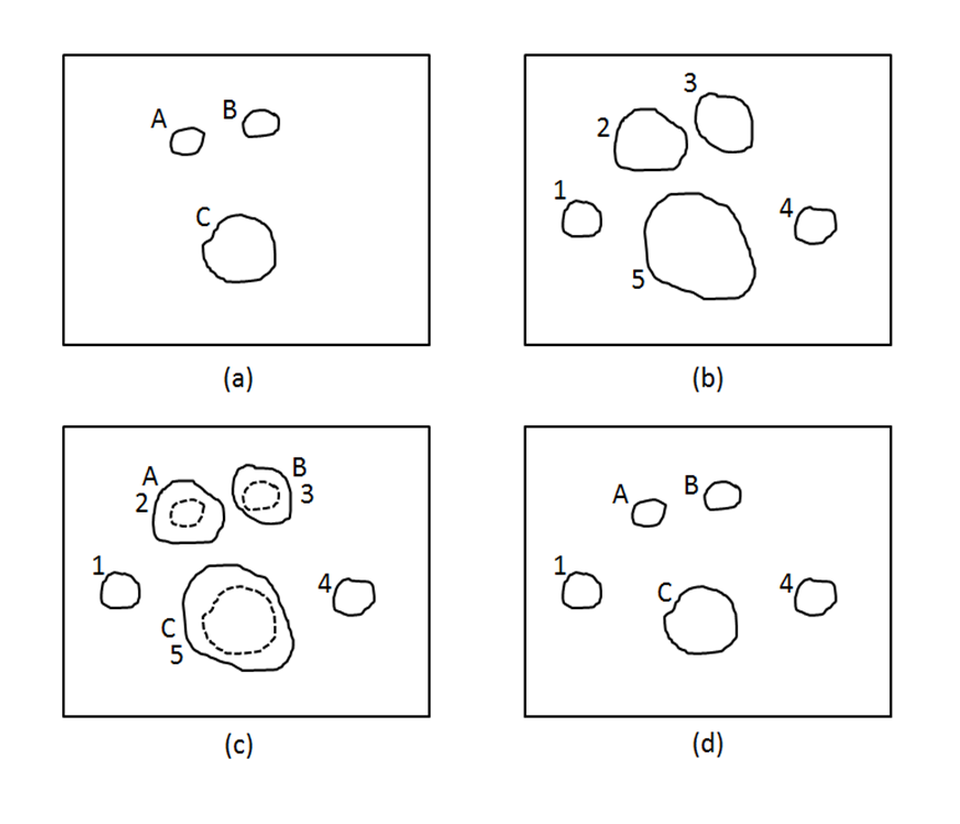

Supplement: S3 Fig — (a) Detected regions at lower sensitivity level; (b) detected regions at higher sensitivity level; (c) overlapping of regions and (d) preserving the regions detected at lower sensitivity level for overlapping regions and obtain final segmentation results. (TIF) [file pone.0142830.s003.tif]

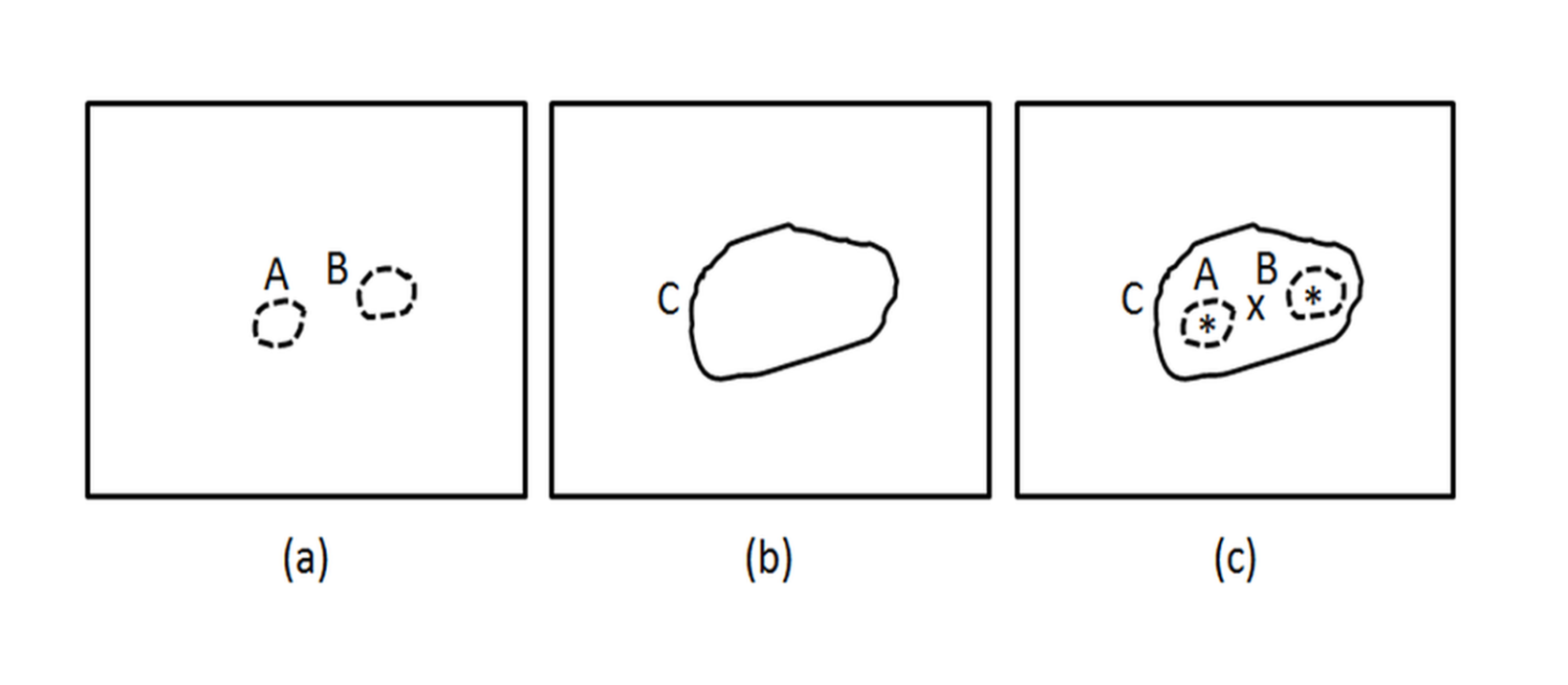

Supplement: S4 Fig — (TIF) [file pone.0142830.s004.tif]

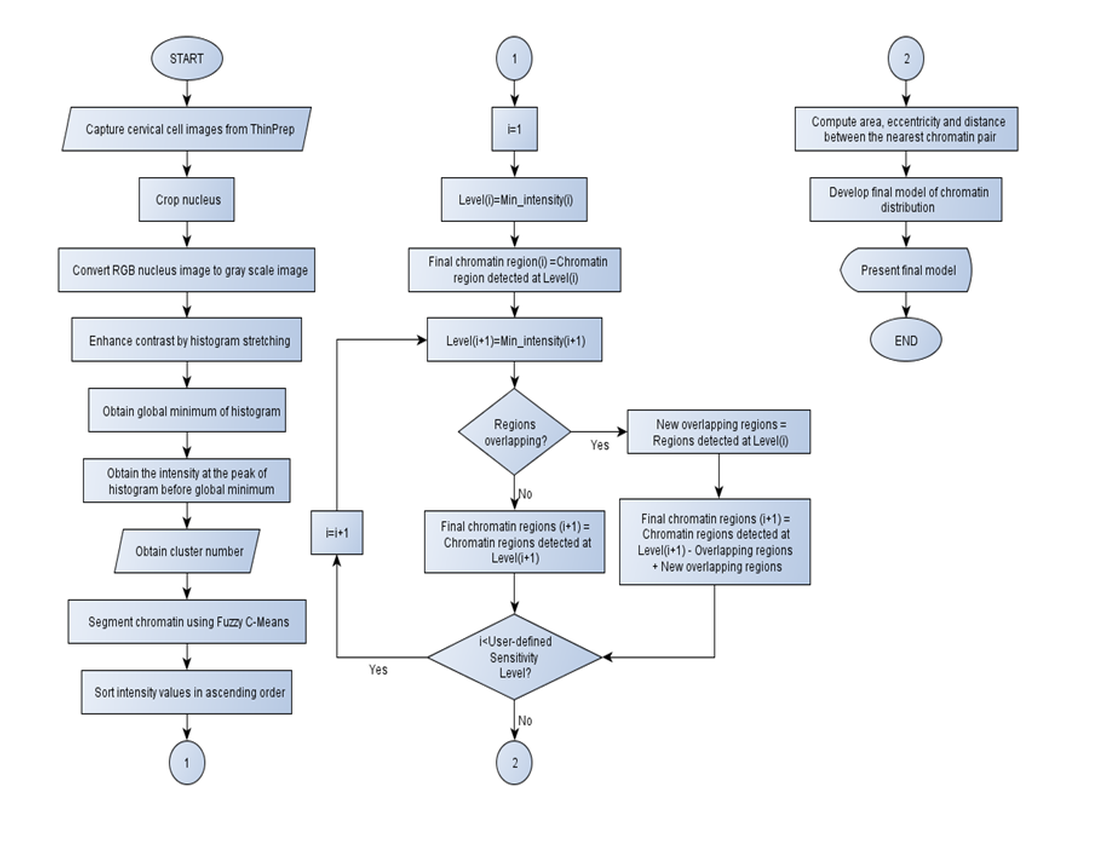

Supplement: S5 Fig — (TIF) [file pone.0142830.s005.tif]
