# Supplementary material for: A Fuzzy-C-Means-Clustering Approach: Quantifying Chromatin Pattern of Non-Neoplastic Cervical Squamous Cells
Source: PLoS One. 2015 Nov 11;10(11):e0142830. doi: 10.1371/journal.pone.0142830 (PMC4641582; doi:10.1371/journal.pone.0142830)
Supplement: S2 File — (DOCX) [file pone.0142830.s007.docx]

S2 File: Defining Cluster Number

The histogram for the cervical nucleus image is constructed to obtain the initial cluster number. The histogram is generally of the bi-modal type since the intensity difference of the nucleus and background is significant. The nucleus is darker than the cytoplasm, and thus the lower part of the histogram separated by the global minimum represents the histogram of the nucleus as demonstrated in Fig. S1. Knowing that the peak of the histogram contains the important information [1,2] , the number of cluster is set to be equal to the intensity which is at the peak of the histogram of the nucleus.

**Fig. S1. The histogram of the image of cropped nucleus.**

The correlation between the sensitivity level and the segmentation threshold can be seen as the contour of the mountain as shown in Fig. S2. As demonstrated in Fig. S2, more chromatin regions can be detected at higher sensitivity level. In other words, only the darker chromatin can be detected at lower sensitivity level. As the sensitivity level increases, the chromatin which appeared brighter can be detected as well. For example, at the 3^rd^ sensitivity level in Fig. S2, new region appeared (i.e. the contour at the lower left part of the image). This indicates that at 1^st^ and 2^nd^ sensitivity level, the intensity threshold is too high to detect the chromatin which appeared bright for these two levels.

**Fig. S2. The sensitivity level and the intensity threshold can be imagined as the contour of a mountain.**

Issues of Overlapping and Combination of Chromatin Regions

As the sensitivity level increases, the chromatin regions detected at the lower sensitivity level will overlap with those detected at higher sensitivity level. The size of the chromatin detected will increase with the increasing of sensitivity levels. From the detected chromatin, the average distance between all the two nearest chromatin pair, the average size (area) of the chromatin and the average eccentricity of the chromatin of each image at each sensitivity level are computed. For each of the chromatin region detected, the centre coordinate value of the region is obtained and used for distance computation.

Since the detected chromatin regions overlap as the sensitivity level increases, the elimination of overlapping regions is performed so that the graphic presentation of the chromatin regions detected will appear to be closer to human eye perception. The elimination process is illustrated in Fig. S3. Figs. S3(a) and (b) show the regions detected at lower and higher sensitivity levels respectively. Regions detected at lower sensitivity level are labelled with alphabet and regions detected for higher sensitivity level are labelled with number.

The overlapping of these chromatin regions at the two sensitivity levels is demonstrated in Fig. S3(c), where regions A and 2 overlap, regions B and 3 overlap and regions C and 5 overlap. Since the overlapping regions represent the same chromatin detected, only the regions at lower sensitivity level are preserved and the final segmentation result is shown in Fig. S3(d). The segmentation result represents the chromatin detected at higher sensitivity level.

**Fig. S3. Overlapping region. (a) Detected regions at lower sensitivity level; (b) detected regions at higher sensitivity level; (c) overlapping of regions and (d) preserving the regions detected at lower sensitivity level for overlapping regions and obtain final segmentation results.**

In addition to the issue of overlapping regions detected along with the increasing number of sensitivity levels, issue of the combination of two detected regions is considered as well. In the case of combination of two or more chromatin regions, instead of taking the entire region and computing its centre, we choose to preserve the chromatin region detected at lower sensitivity level. This makes the detection results appeared closer to human eye perception. Consequently, this avoids the scenario demonstrated in Fig. S4. Fig. S4(a) shows the detection of two chromatin regions A and B at low sensitivity level while Fig. S4(b) shows the detection of chromatin region C at the same position at a higher sensitivity level as compared to Fig. S4(a). In such case, the centre of the chromatin closer to human eyesight should be those marking with asterisk, “*” rather than the cross, “x”. Therefore, for the overlapping chromatin regions at higher sensitivity level, regions A and B are retained while region C is eliminated.

The overview of the chromatin segmentation process is illustrated in Fig. S5.

**Fig. S4. Combination of two chromatin regions: (a) Chromatin regions at lower sensitivity level; (b) Chromatin region at higher sensitivity level and (c) The centres of the chromatin regions when regions A, B and C overlap.**

**Fig. S5. Flowchart of chromatin segmentation.**

References

**1.** Heng-Da C, Ying S (2000) A hierarchical approach to color image segmentation using homogeneity. IEEE Trans. on Imag. Process. 9: 2071-2082.

**2.** Yen-Ching C, Chun-Ming C (2010) A simple histogram modification scheme for contrast enhancement. IEEE Trans. on Consum. Electron. 56: 737-742.
