# Supplementary material for: A Fuzzy-C-Means-Clustering Approach: Quantifying Chromatin Pattern of Non-Neoplastic Cervical Squamous Cells
Source: PLoS One. 2015 Nov 11;10(11):e0142830. doi: 10.1371/journal.pone.0142830 (PMC4641582; doi:10.1371/journal.pone.0142830)
Supplement: S3 File — (DOCX) [file pone.0142830.s008.docx]

S3 File: Parameter Testing for Amount of Fuzziness

Parameter testing has been performed on the amount of fuzziness,$m$. Statistical analyses have been performed for$m=\left\{1.2,3,4\right\}$at every sensitivity level. The *p*-value returned for the Friedman test at α=0.05 for the analysis of the average area and average distance of the nearest chromatin pair are shown in Table S1. All *p*-values are less than α for both the average area and average distance of the nearest chromatin pair, indicating that at different amount of fuzziness, the hypothesis on no differences between the sensitivity levels is rejected. Hence, Post hoc test is carried out to investigate which sensitivity levels differ from which other sensitivity levels. Results of the post hoc analysis with Holm’s and Shaffer’s procedures for the average area of chromatin, with different amount of fuzziness, are shown in Tables S2 to S5, and for the average distance of the nearest chromatin pair are shown in Tables S6 to S9. For all the analyses, *p*-values that are not less than 0.05 are written in boldface, indicating that at these sensitivity levels, there is no significant difference for the measurement, either for the average area or for the average distance of the nearest chromatin pair. Summary of Tables S2 to S9 is presented in Table 2.

**Table S1. *p-values* of Friedman Test.**

| Amount of Fuzziness, $m$ | Average Area of Chromatin  ($\times10^{-10}$) | Average Distance of the Nearest Chromatin Pair($\times10^{-10}$) |
| --- | --- | --- |
| 1.2 | 1.251 | 1.076 |
| 2.0 | 1.856 | 1.912 |
| 3.0 | 2.045 | 1.654 |
| 4.0 | 2.152 | 1.698 |

**Table S2. Adjusted p-values for N × N comparisons of five sensitivity levels for the area of chromatin with m=1.2.**

| Level vs. Level | Holm | Shaffer |
| --- | --- | --- |
| [1] vs. [5] | 0.005000 | 0.005000 |
| [1] vs. [4] | 0.005556 | 0.008333 |
| [1] vs. [3] | 0.008333 | 0.008333 |
| [1] vs. [2] | 0.016667 | 0.016667 |
| [2] vs. [5] | 0.006250 | 0.008333 |
| [2] vs. [4] | 0.007143 | 0.008333 |
| [2] vs. [3] | 0.025000 | 0.025000 |
| [3] vs. [5] | 0.010000 | 0.012500 |
| [3] vs. [4] | 0.012500 | 0.012500 |
| [4] vs. [5] | **0.050000** | **0.050000** |

**Table S3. Adjusted p-values for N × N comparisons of five sensitivity levels for the area of chromatin with m=2.0.**

| Level vs. Level | Holm | Shaffer |
| --- | --- | --- |
| [1] vs. [5] | 0.005000 | 0.005000 |
| [1] vs. [4] | 0.005556 | 0.008333 |
| [1] vs. [3] | 0.008333 | 0.008333 |
| [1] vs. [2] | 0.016667 | 0.016667 |
| [2] vs. [5] | 0.006250 | 0.008333 |
| [2] vs. [4] | 0.007143 | 0.008333 |
| [2] vs. [3] | 0.025000 | 0.025000 |
| [3] vs. [5] | 0.010000 | 0.012500 |
| [3] vs. [4] | 0.012500 | 0.012500 |
| [4] vs. [5] | **0.050000** | **0.050000** |

**Table S4. Adjusted p-values for N × N comparisons of five sensitivity levels for the area of chromatin with m=3.0.**

| Level vs. Level | Holm | Shaffer |
| --- | --- | --- |
| [1] vs. [5] | 0.005000 | 0.005000 |
| [1] vs. [4] | 0.005556 | 0.008333 |
| [1] vs. [3] | 0.007143 | 0.008333 |
| [1] vs. [2] | 0.016667 | 0.016667 |
| [2] vs. [5] | 0.006250 | 0.008333 |
| [2] vs. [4] | 0.008333 | 0.008333 |
| [2] vs. [3] | 0.012500 | 0.012500 |
| [3] vs. [5] | 0.010000 | 0.012500 |
| [3] vs. [4] | 0.025000 | 0.025000 |
| [4] vs. [5] | **0.050000** | **0.050000** |

**Table S5. Adjusted p-values for N × N comparisons of five sensitivity levels for the area of chromatin with m=4.0.**

| Level vs. Level | Holm | Shaffer |
| --- | --- | --- |
| [1] vs. [5] | 0.005000 | 0.005000 |
| [1] vs. [4] | 0.005556 | 0.008333 |
| [1] vs. [3] | 0.007143 | 0.008333 |
| [1] vs. [2] | 0.012500 | 0.012500 |
| [2] vs. [5] | 0.006250 | 0.008333 |
| [2] vs. [4] | 0.008333 | 0.008333 |
| [2] vs. [3] | 0.016667 | 0.016667 |
| [3] vs. [5] | 0.010000 | 0.012500 |
| [3] vs. [4] | 0.025000 | 0.025000 |
| [4] vs. [5] | **0.050000** | **0.050000** |

**Table S6. Adjusted p-values for N × N comparisons of five sensitivity levels for the distance between nearest chromatin pair with m=1.2.**

| Level vs. Level | Holm | Shaffer |
| --- | --- | --- |
| [1] vs. [5] | 0.005000 | 0.005000 |
| [1] vs. [4] | 0.005556 | 0.008333 |
| [1] vs. [3] | 0.007143 | 0.008333 |
| [1] vs. [2] | 0.012500 | 0.012500 |
| [2] vs. [5] | 0.006250 | 0.008333 |
| [2] vs. [4] | 0.008333 | 0.008333 |
| [2] vs. [3] | 0.016667 | 0.016667 |
| [3] vs. [5] | 0.010000 | 0.012500 |
| [3] vs. [4] | **0.050000** | **0.050000** |
| [4] vs. [5] | 0.025000 | 0.025000 |

**Table S7. Adjusted p-values for N × N comparisons of five sensitivity levels for the distance between nearest chromatin pair with m=2.0.**

| Level vs. Level | Holm | Shaffer |
| --- | --- | --- |
| [1] vs. [5] | 0.005000 | 0.005000 |
| [1] vs. [4] | 0.005556 | 0.008333 |
| [1] vs. [3] | 0.007143 | 0.008333 |
| [1] vs. [2] | 0.012500 | 0.012500 |
| [2] vs. [5] | 0.006250 | 0.008333 |
| [2] vs. [4] | 0.008333 | 0.008333 |
| [2] vs. [3] | 0.025000 | 0.025000 |
| [3] vs. [5] | 0.010000 | 0.012500 |
| [3] vs. [4] | 0.025000 | 0.025000 |
| [4] vs. [5] | **0.050000** | **0.050000** |

**Table S8. Adjusted p-values for N × N comparisons of five sensitivity levels for the distance between nearest chromatin pair with m=3.0.**

| Level vs. Level | Holm | Shaffer |
| --- | --- | --- |
| [1] vs. [5] | 0.005000 | 0.005000 |
| [1] vs. [4] | 0.005556 | 0.008333 |
| [1] vs. [3] | 0.007143 | 0.008333 |
| [1] vs. [2] | 0.012500 | 0.012500 |
| [2] vs. [5] | 0.006250 | 0.008333 |
| [2] vs. [4] | 0.008333 | 0.008333 |
| [2] vs. [3] | 0.016667 | 0.016667 |
| [3] vs. [5] | 0.010000 | 0.012500 |
| [3] vs. [4] | 0.025000 | 0.025000 |
| [4] vs. [5] | **0.050000** | **0.050000** |

**Table S9. Adjusted p-values for N × N comparisons of five sensitivity levels for the distance between nearest chromatin pair with m=4.0.**

| Level vs. Level | Holm | Shaffer |
| --- | --- | --- |
| [1] vs. [5] | 0.005000 | 0.005000 |
| [1] vs. [4] | 0.005556 | 0.008333 |
| [1] vs. [3] | 0.007143 | 0.008333 |
| [1] vs. [2] | 0.016667 | 0.016667 |
| [2] vs. [5] | 0.006250 | 0.008333 |
| [2] vs. [4] | 0.008333 | 0.008333 |
| [2] vs. [3] | 0.012500 | 0.012500 |
| [3] vs. [5] | 0.010000 | 0.012500 |
| [3] vs. [4] | 0.025000 | 0.025000 |
| [4] vs. [5] | **0.050000** | **0.050000** |
