# Supplementary material for: A Fuzzy-C-Means-Clustering Approach: Quantifying Chromatin Pattern of Non-Neoplastic Cervical Squamous Cells
Source: PLoS One. 2015 Nov 11;10(11):e0142830. doi: 10.1371/journal.pone.0142830 (PMC4641582; doi:10.1371/journal.pone.0142830)
Supplement: S4 File — (DOCX) [file pone.0142830.s009.docx]

S4 File: Complexity Analysis of FCM

Complexity of FCM in segmenting the chromatin is verified through computational time. The average computational time for 150 test images at each sensitivity level with different amount of fuzziness is measured under the same platform and the results are tabulated as in Table S10. It can be seen that the average computational time is similar for each sensitivity level at different amount of fuzziness, with mean computational time per iteration of 0.8 seconds. Therefore, it can be concluded that factors of sensitivity level and amount of fuzziness do not affect the complexity of the algorithm.

**Table S10. Average computational time for 150 test images.**

| m | Level | | | | | Mean Computational Time (i.e. For 100 Iterations) (s) | Mean Computational Time (i.e. For 1 Iteration) (s) |
| --- | --- | --- | --- | --- | --- | --- | --- |
|  | 1 | 2 | 3 | 4 | 5 |  |  |
| 1.2 | 83.506 | 79.731 | 83.721 | 84.378 | 79.002 | 82.068 | 0.821 |
| 2.0 | 83.043 | 81.532 | 82.310 | 84.859 | 83.336 | 83.016 | 0.830 |
| 3.0 | 81.519 | 84.699 | 83.490 | 84.914 | 85.173 | 83.959 | 0.840 |
| 4.0 | 79.487 | 79.343 | 77.562 | 77.640 | 78.007 | 78.408 | 0.784 |
