# Supplementary material for: A Fuzzy-C-Means-Clustering Approach: Quantifying Chromatin Pattern of Non-Neoplastic Cervical Squamous Cells
Source: PLoS One. 2015 Nov 11;10(11):e0142830. doi: 10.1371/journal.pone.0142830 (PMC4641582; doi:10.1371/journal.pone.0142830)
Supplement: S1 Table — (DOCX) [file pone.0142830.s010.docx]

**Table S1. p-values of Friedman Test.**

| **Amount of Fuzziness, $m$** | **Average Area of Chromatin**  **($\times10^{-10}$)** | **Average Distance of the Nearest Chromatin Pair($\times10^{-10}$)** |
| --- | --- | --- |
| **1.2** | **1.251** | **1.076** |
| **2.0** | **1.856** | **1.912** |
| **3.0** | **2.045** | **1.654** |
| **4.0** | **2.152** | **1.698** |
