# Supplementary material for: A Fuzzy-C-Means-Clustering Approach: Quantifying Chromatin Pattern of Non-Neoplastic Cervical Squamous Cells
Source: PLoS One. 2015 Nov 11;10(11):e0142830. doi: 10.1371/journal.pone.0142830 (PMC4641582; doi:10.1371/journal.pone.0142830)
Supplement: S7 Table — (DOCX) [file pone.0142830.s016.docx]

**Table S7. Adjusted p-values for N × N comparisons of five sensitivity levels for the distance between nearest chromatin pair with m=2.0.**

| **Level vs. Level** | **Holm** | **Shaffer** |
| --- | --- | --- |
| **[1] vs. [5]** | **0.005000** | **0.005000** |
| **[1] vs. [4]** | **0.005556** | **0.008333** |
| **[1] vs. [3]** | **0.007143** | **0.008333** |
| **[1] vs. [2]** | **0.012500** | **0.012500** |
| **[2] vs. [5]** | **0.006250** | **0.008333** |
| **[2] vs. [4]** | **0.008333** | **0.008333** |
| **[2] vs. [3]** | **0.025000** | **0.025000** |
| **[3] vs. [5]** | **0.010000** | **0.012500** |
| **[3] vs. [4]** | **0.025000** | **0.025000** |
| **[4] vs. [5]** | **0.050000** | **0.050000** |
