# Supplementary material for: A Fuzzy-C-Means-Clustering Approach: Quantifying Chromatin Pattern of Non-Neoplastic Cervical Squamous Cells
Source: PLoS One. 2015 Nov 11;10(11):e0142830. doi: 10.1371/journal.pone.0142830 (PMC4641582; doi:10.1371/journal.pone.0142830)
Supplement: S10 Table — (DOCX) [file pone.0142830.s019.docx]

**Table S10. Average computational time for 150 test images.**

| m | Level | | | | | Mean Computational Time (i.e. For 100 Iterations) (s) | Mean Computational Time (i.e. For 1 Iteration) (s) |
| --- | --- | --- | --- | --- | --- | --- | --- |
|  | 1 | 2 | 3 | 4 | 5 |  |  |
| 1.2 | 83.506 | 79.731 | 83.721 | 84.378 | 79.002 | 82.068 | 0.821 |
| 2.0 | 83.043 | 81.532 | 82.310 | 84.859 | 83.336 | 83.016 | 0.830 |
| 3.0 | 81.519 | 84.699 | 83.490 | 84.914 | 85.173 | 83.959 | 0.840 |
| 4.0 | 79.487 | 79.343 | 77.562 | 77.640 | 78.007 | 78.408 | 0.784 |
